# Fatal anaphylactic shock due to hymenoptera venom in a farmer suffering from indolent systemic mastocytosis. The comparative diagnostic relevance of perimortem serum tryptase levels

**DOI:** 10.1007/s00414-025-03487-1

**Published:** 2025-04-02

**Authors:** Ugo Da Broi, Francesco Simonit, Maurizio Perogio, Daniela Visentini, Federico Reccardini, Rexson Tse, Jack Garland, Benjamin Ondruschka, Lorenzo Desinan

**Affiliations:** 1 Department of Medicine, Legal Medicine, University of Udine, Udine, Italy; 2 Department of Medical Surgical and Health Sciences, School of Legal Medicine, University of Trieste, Trieste, Italy; 3 Department of Clinical Pathology, Immuno-Allergology Lab, SMM General Hospital, Udine, Italy; 4 Department of Pulmonology, SMM General Hospital, Udine, Italy; 5 Griffith University School of Medicine and Dentistry, Southport, QLD Australia; 6 Queensland Public Health and Scientific Services, Brisbane, QLD Australia; 7 Institute of Legal Medicine, University Medical Center Hamburg-Eppendorf, Hamburg, Germany

**Keywords:** Anaphylaxis, Anaphylactic death, Hymenoptera venom, Indolent systemic mastocytosis, Serum tryptase

## Abstract

Hymenoptera anaphylaxis led to the death of a bee and wasp venom sensitized 41-year-old man suffering from systemic indolent mastocytosis. While at work in a vineyard, the man suffered a serious anaphylactic crisis and cardiovascular arrest; despite ongoing attempts of resuscitation, he died in hospital 12 h after being stung. Autopsy confirmed that death was due to post-anoxic brain damage, cardiovascular shock, disseminated intravascular coagulation (DIC) and multi-organ failure (MOF). ICU blood samples drawn before the patient’s death from the distal extremity of the pulmonary catheter revealed central blood tryptase levels of 8955 ng/mL; samples drawn 6 days after death, at autopsy, confirmed anaphylaxis diagnostic central blood total tryptase levels (4977 ng/mL) and peripheral blood levels (319 ng/mL); IgE levels in ICU blood sample suggested that the farmer was a responder to venom immunoteraphy (VIT) for *Apis Mellifera* (IgE 0.44 kUI/L) but not for *Polistes Dominulus* (IgE 3.13 kUI/L) yet. The comparison of perimortem laboratory results was crucial, in association with autopsy findings and circumstantial data, in ascertaining that death was caused by a wasp venom anaphylactic reaction, with key findings being: 1) Significantly high pre-mortem (8955 ng/mL) and post-mortem (4977 ng/mL) central blood tryptase levels. 2) High post-mortem peripheral blood tryptase levels (319 ng/mL). 3) High pre-mortem central blood IgE antibodies against *Polistes Dominulus*.

## Introduction

Anaphylaxis is a life-threatening condition resulting from the immunoglobulin IgE-mediated release of vasoactive and inflammatory compounds from mast cells and basophils, which occurs after the interaction of specific allergenic antigens (drugs, foods, hymenoptera venoms, contrast reagents and allergen extracts) and IgE in previously sensitized patients, with the involvement of two or more body systems, typically skin-mucosal tissue and the respiratory, cardiovascular and gastrointestinal systems [1, 2].

Pruritus, urticaria pigmentosa, wheezing, bronchospasm, hypotension, vomiting and diarrhoea are typical symptoms, while victims suffering from serious, rapid cardiorespiratory impairment are at risk of death, with frequencies ranging from 0.12 to 1.06 deaths per million every year worldwide [1–3].

Hymenoptera venom anaphylaxis has been claimed to affect 0.3 up to 8.9% of all adults stung, with worldwide mortality ranging from 0.03 to 0.48 cases per million a year, while 20–30% of all cases of anaphylaxis affecting the general population involves patients with mastocytosis et al.^4,5^ Mastocytosis is a proliferative disorder of the haematopoietic system which affects mast cell progenitors and results in an accumulation of excessive numbers of mast cells in a variety of tissues (such as bone marrow, skin, mucosae, liver, gastrointestinal tract, spleen and lymph nodes) and is characterized by the presence of:

- Clinical signs involving the skin (urticaria pigmentosa, angioedema, flushing), the gastrointestinal system (nausea, vomiting, diarrhea, abdominal cramping), the cardiovascular system (tachycardia, hypotension, syncope) or the upper and lower respiratory system (conjunctival injection, nasal pruritus, nasal congestion, wheezing, dyspnea) [4–8].
- Substantial elevation of basic serum tryptase levels (> 20ng/mL), and response to mast cell mediator therapy (antihistamines, steroids and leukotriene blockers) [7].

Various published reports underline the fact that subjects suffering from mastocytosis, when sensitized to allergens, especially to wasp venom, drugs, contrast media, foods, pollens, and vaccines, are at high risk of severe anaphylactic shock; this is essentially caused by the over-proliferation (through KIT816V gene mutation) and dysfunction (through gene TPSAB1 mutation and the resulting production of tryptase) of mast cells which may also display a genetically-based overexpression and/or malfunctioning of various receptors such as MRGPRX2 (inducing non immunoglobulin IgE-mediated degranulation and release of anaphylactic compounds), FcεgE (main site of interaction with immunoglobulin IgE) and CD2,CD25,CD30,CD45, CD117 [5, 9–19].

Indolent systemic mastocytosis is the most common variant and affects more than 80% of all patients suffering from systemic mastocytosis. It is typically present in younger adults with a high prevalence of skin issues (urticaria pigmentosa), hepatomegaly and with baseline tryptase levels > 125 ng/mL [7, 20, 21].

Adult male patients suffering from indolent systemic mastocytosis (especially in cases without previous skin issues) are at extremely high risk of severe and recurrent systemic reactions to hymenoptera stings. These can be life threatening in 90% of cases of mastocytosis-affected subjects who are not protected by immunotheraphy, who are not carrying an epinephrine auto injector and who may display basic hyperreactivity, described as both spontaneous or consequent to exogenous triggers (IgE or sometimes not IgE mediated) [9, 10, 14–19, 22, 23].

It was also reported that the coincidence of basic cardiovascular ischaemic comorbidities with an anaphylactic event, including those triggered by hymenoptera venom, may seriously impact the cardiovascular system and lead to the dangerous sting-related Kounis syndrome [9, 10, 22–25].

We report and describe the medico-legal investigations in a case of fatal anaphylactic shock due to hymenoptera venom in a farmer suffering from indolent systemic mastocytosis and discuss the comparative diagnostic relevance of perimortem serum tryptase levels.

## Case report

A bee and wasp venom sensitized 41-year-old man working in a vineyard, was stung by a hymenopteran, became dyspnoeic and collapsed and although his mother, who was quickly on the scene, injected him with epinephrine with an auto-injector, he shortly lost consciousness and went into cardiac arrest.

Due to the remote location, it was 20 min before an emergency team arrived at the site by helicopter. They found the man in cardiovascular arrest and in a state of pulseless electrical activity (PEA). The rescue team immediately began cardiopulmonary resuscitation (CPR), performed orotracheal intubation, administered 9 mg of adrenaline intravenously and helicoptered the man to hospital, while performing ongoing external cardiac massage with automated chest compression device (ACCD - LUCAS instrumentation). On admission he was immediately transferred to the intensive care unit (ICU) where he was put on an ECMO device. The ACCD was kept working for a total time of 1 h and 20 min from the arrival of the rescue team at the vineyard. The patient also underwent intravenous administration of epinephrine and norepinephrine in addition to calcium gluconate, NaHCO3, glucose and insulin to correct severe acidosis with hyperkalemia. Hydrocortisone, chlorphenamine, albumin and 2000 mL of crystalloids were also administered. The patient was, however, haemodynamically unstable, with his blood pressure being maintained only by high-dose inotropes and vasopressors in continuous infusion.

A computed tomography brain scan revealed a widespread lack of differentiation between white matter and cortical and deep gray matter, compatible with severe post-anoxic brain oedema and coma. During the hours following hospital admission serious metabolic acidosis set in, with low cardiac output syndrome and haemodynamic instability which were unresponsive to treatment with inotropic amines and fluids. This gradually evolved into disseminated intravascular coagulation (DIC) and multi-organ failure (MOF). Despite resuscitative treatments the subject died 12 h after being stung (Table 1).

The autopsy, performed 6 days after death (as directed by the Public Prosecutor’s Office), identified the following:

**Table 1:** Main autopsy findings. Histological analysis showed

Sites	Findings
Brain	• Cerebral oedema • Weight: 1740 g
Larynx-Trachea	• Vocal cords oedema (Fig. 1) • Tracheal mucosal oedema (Fig. 1)
Lungs	• severe congestion and oedema • weight: right 720 g, left 740 g
Heart	• pale with dilated chambers • normal coronaries • weight 420 g
Abdominal organs	• Empty stomach, with autolytic mucosa showing haemorrhagic specks • Duodenum: no lesions • Pancreas: lobulated • Liver: increased in weight (1720 g) and volume, and congested • Spleen (240 gr): congested, with retained pulp
Urogenital system	• Right and left kidney (both 120 g): smooth surface, with attenuated cortico-medullary contrast • bladder with whitish mucosa; normotrophic prostate
Endocrine system	• Adrenal: putrefied medulla

**Table 2:** Main histological findings

Sites	Findings
Brainstem	• Congestion and regressive alterations
Vocal cords	• Focal inflammation (Figs. 2 and 3)
Bronchus	• No lesions
Lungs	• Congestion and oedema • Swollen nodules (Figs. 4 and 5)
Bone marrow	• Hyperplastic mixed cellularity
Skin	• Chronic inflammatory infiltrates of chorion
Myocardium	• Fresh contraction bands
Spleen	• Congestion • Poor representation of white pulp
Liver	• Polyfocal steatosis with small and large drops • Chronic inflammatory infiltration of the portal spaces
Kidneys	• Diffuse tubulonephrosis (Fig. 6)

**Fig. 1:**
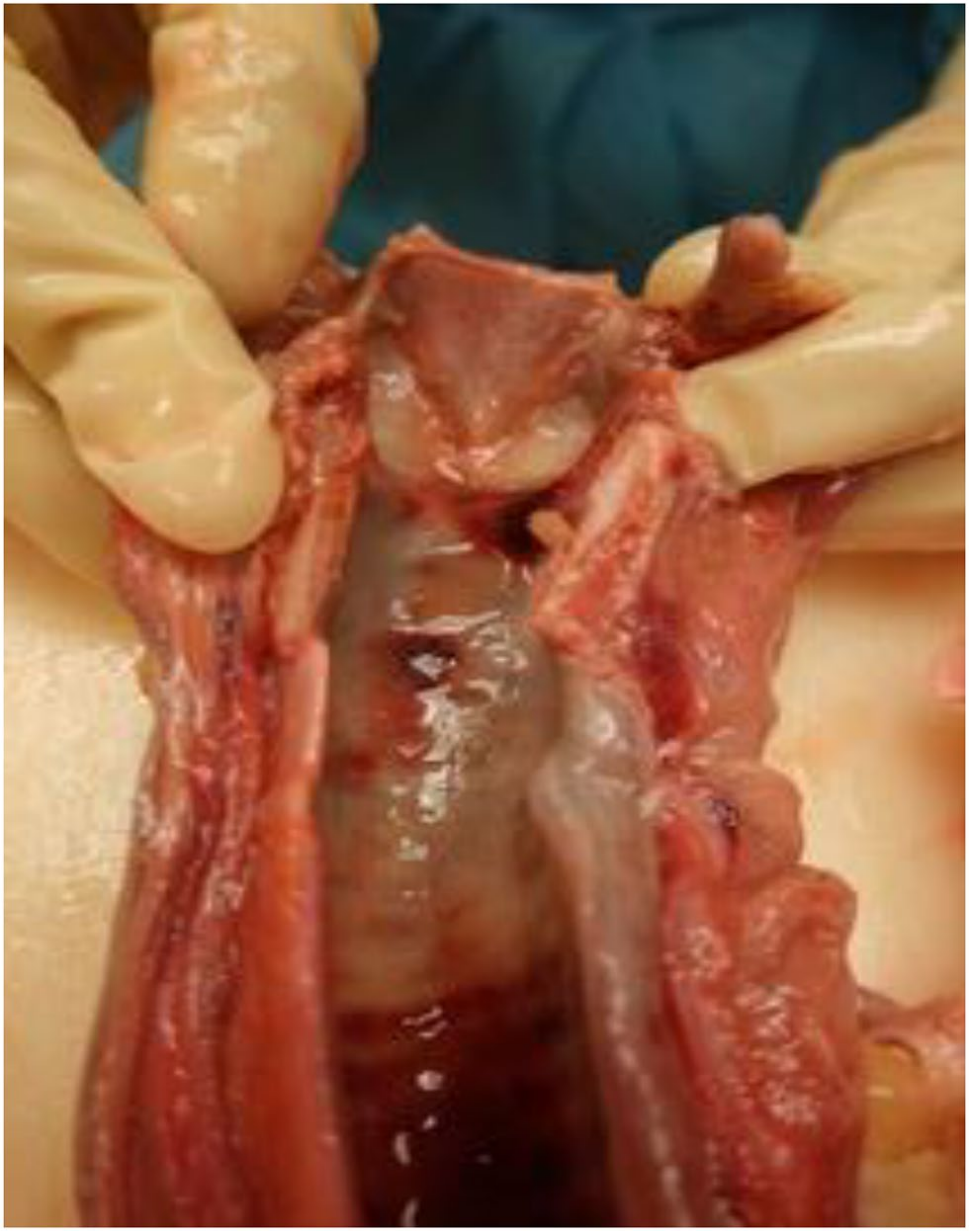
Vocal cords oedema - Tracheal mucosal oedema

**Fig. 2:**
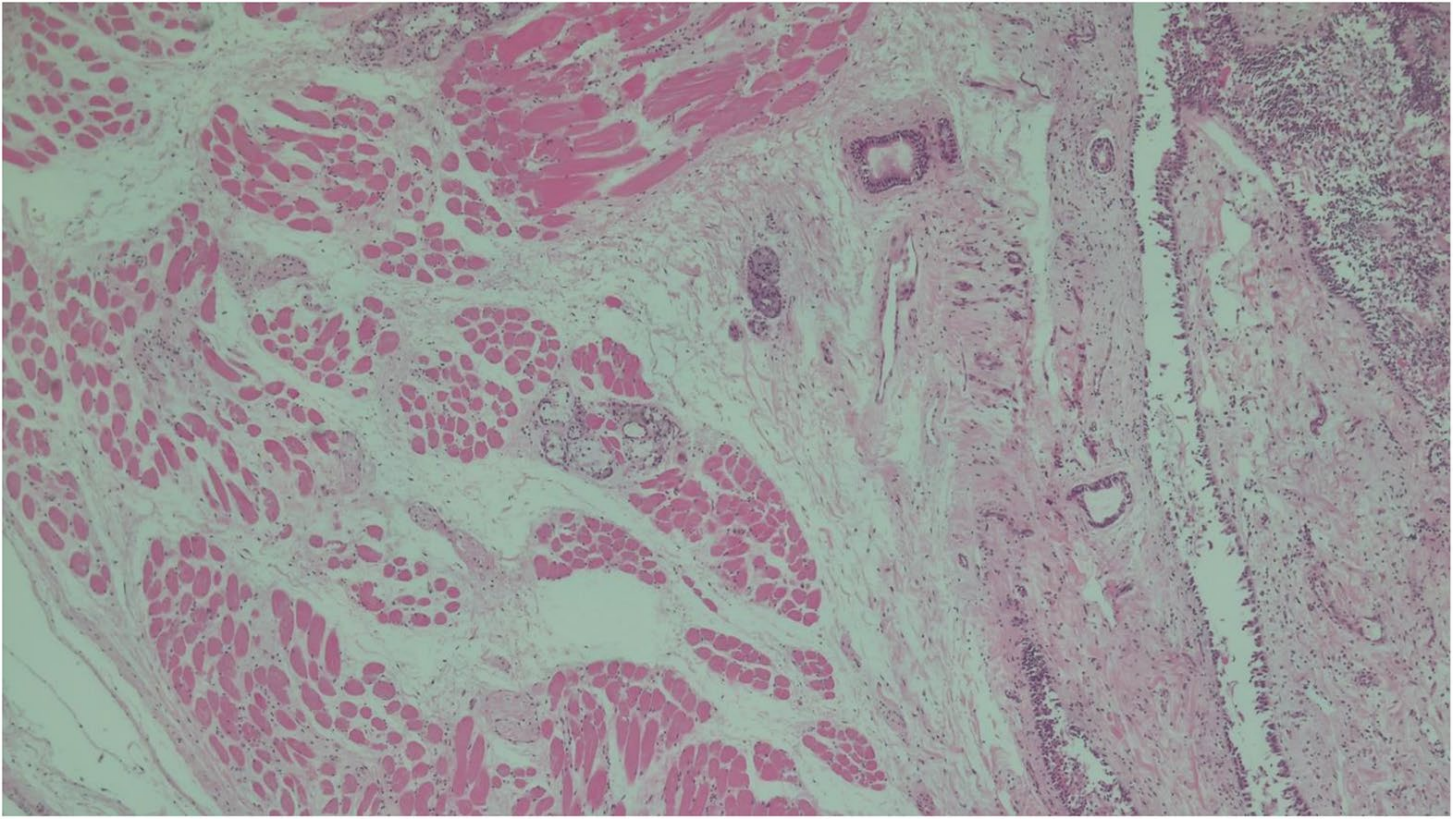
Vocal cord (hematoxylin eosin, 5x) - Focal inflammation

**Fig. 3:**
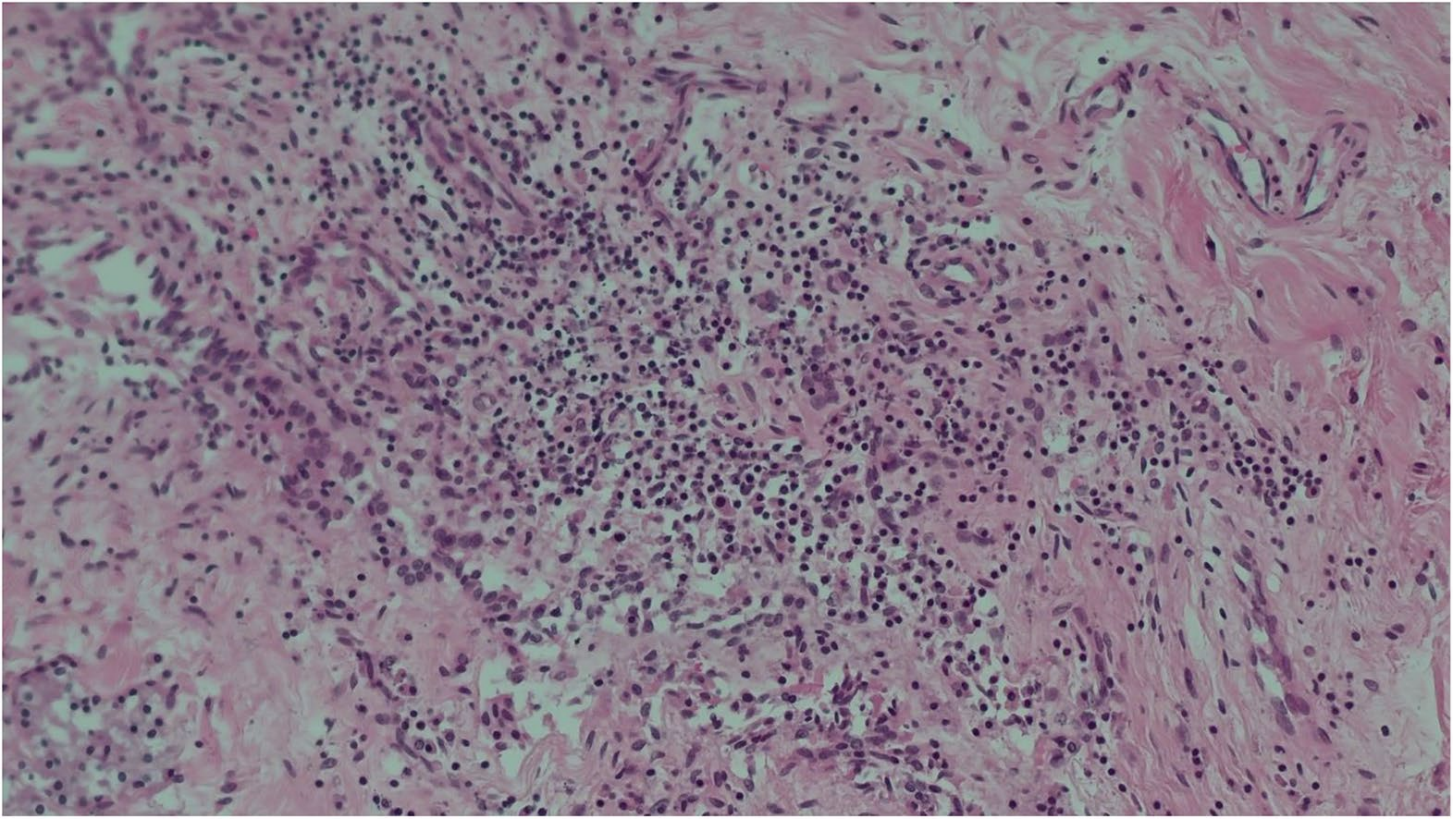
Vocal cord (hematoxylin eosin, 20x) - Focal inflammation

**Fig. 4:**
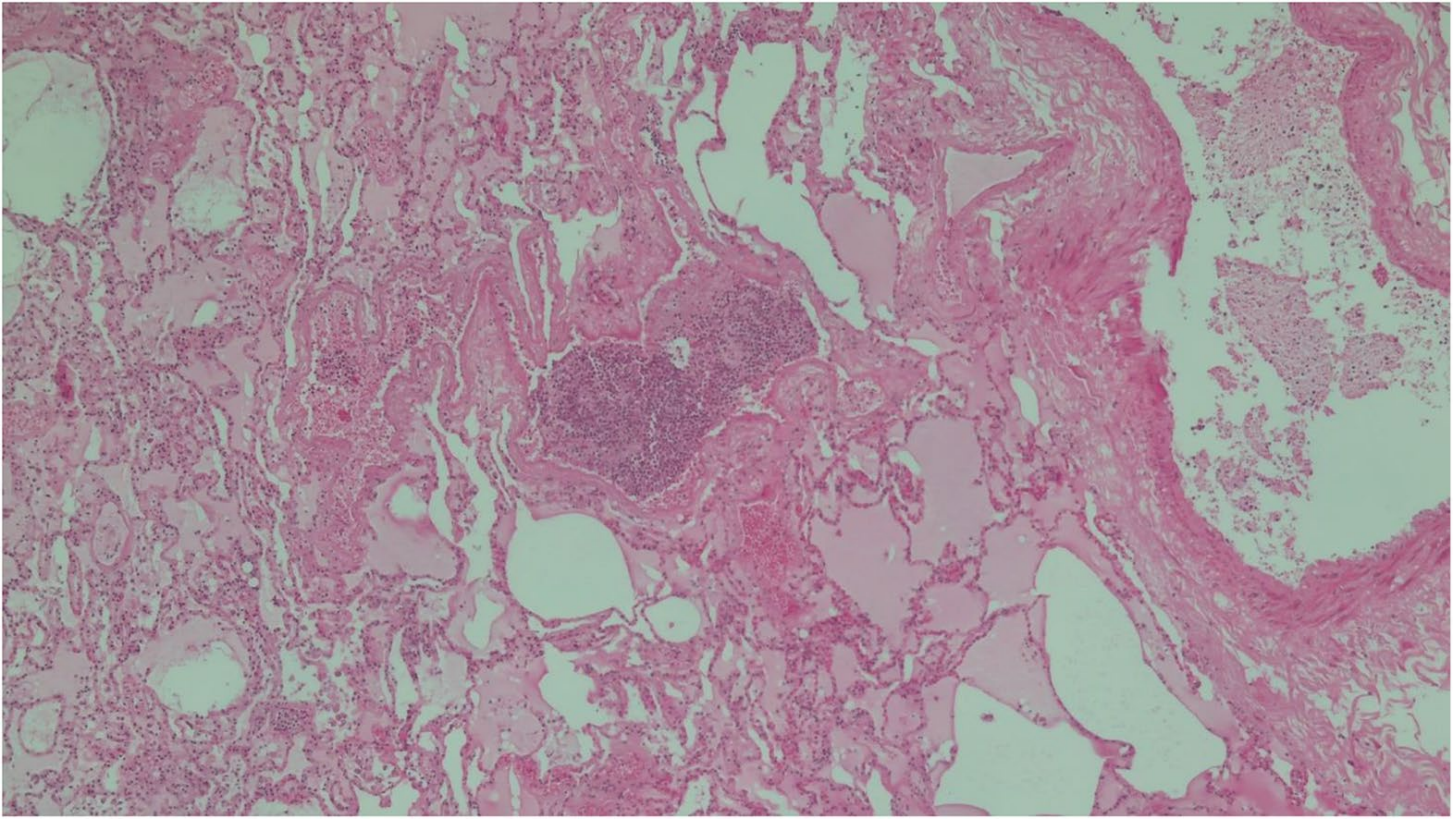
Lung (hematoxylin eosin, 5x) - Congestion, oedema and phlogistic nodule

**Fig. 5:**
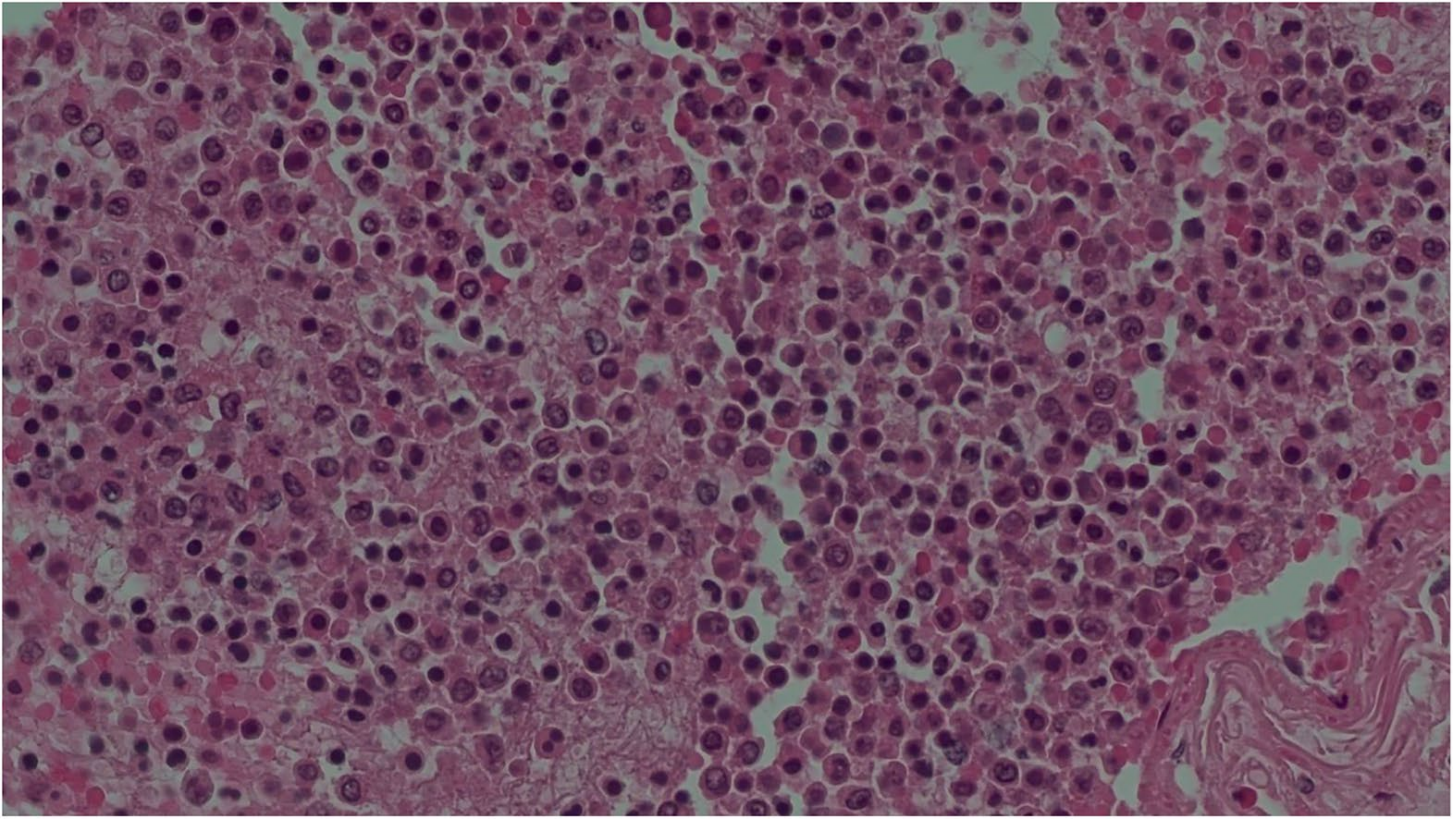
Lung (hematoxylin eosin, 40x) - Phlogistic nodule with eosinophils

**Fig. 6:**
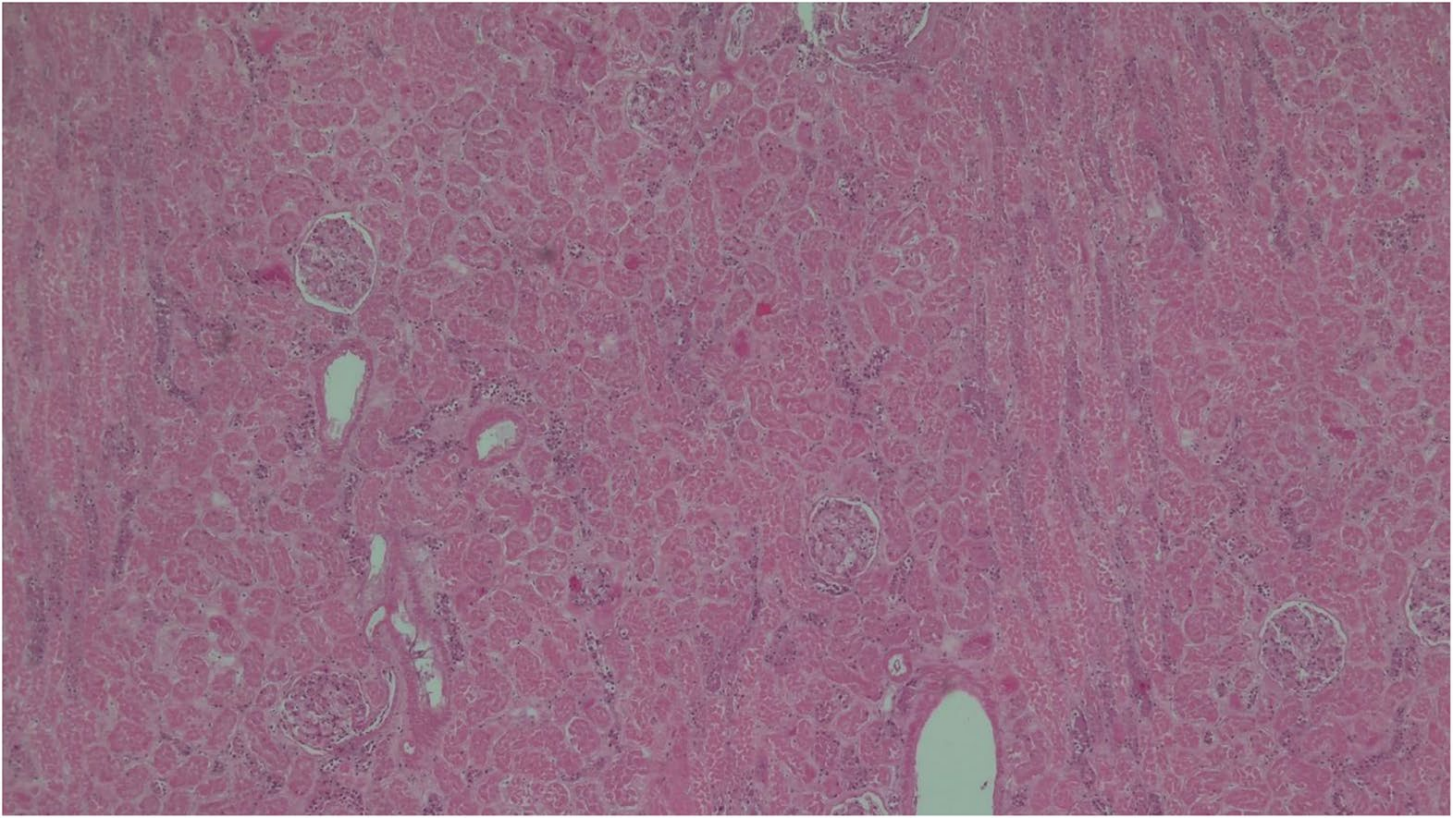
Kidney (hematoxylin eosin, 5x) - Diffuse tubulonephrosis

Based on clinical history, autopsy results and histological data, although the absence of cutaneous signs compatible with a hymenoptera sting, the cause of death was attributed to anaphylactic shock secondary to a wasp sting and indolent systemic mastocytosis which was a significant contributing factor (Table 2).

An investigation of the victim’s medical history showed that he was suffering from indolent systemic mastocytosis with sensitization to *Apis mellifera* and *Polistes Dominulus* venom, with the presence of serum specific IgE levels for these two different Hymenoptera venoms (see pre-mortem IgE levels in ICU blood samples - Table 3). The individual had previously been screened at an allergology centre because he had already been stung several times by hymenoptera, but without serious cardiovascular consequences. When he died, he was in the process of undergoing desensitization therapy for hymenoptera venom, with a total of 8 venom immunotherapy (VIT) sessions carried out over 21 months (7 sessions with specific *Apis mellifera* venom and one session with specific *Polistes Dominulus* venom).

During resuscitative procedures in the ICU, a central blood sample from a pulmonary catheter was taken 5 h after the onset of cardiac arrest to perform routine laboratory analysis. After being stored in the laboratory (in anticipation of a potential organ donation), it was used for post-mortem research into IgE (Immunofluorimetric assay FEIA ThermoFisher Scientific - Phadia 2500 Instrument) and tryptase (Immunofluorimetric assay FEIA ThermoFisher Scientific - Phadia 250 Instrument) levels, which had diagnostic values (see tryptase levels in Table 4).

Following the victim’s death, central and peripheral blood samples collected at autopsy were analyzed in order to identify post-mortem tryptase levels, which were also of diagnostic value (see tryptase levels in Table 4).

**Table 3:** Pre-mortem IgE levels (ICU blood samples)

Matrix	IgE (No sensitization values < 0.15 kUI/L)
Peripheral blood	0.44 - *Apis Mellifera*
Peripheral blood	3.13 - *Polistes Dominulus (European Paper Wasp)*

**Table 4:** Post-mortem (autopsy performed 6 days after death) and pre-mortem (ICU blood samples) serum tryptase levels

Matrix	Serum tryptase (clinical normal values < 15–20 ng/mL) (post-mortem values < 23 ng/mL)^27,36,40^
Central blood pre-mortem (from pulmonary catheter - ICU)	8955
Central blood post-mortem	4977
Peripheral blood post-mortem	319
Peripheral blood pre-mortem (one year before death -for hematological surveillance of systemic indolent mastocytosis)	165
Peripheral blood pre-mortem (two years before death -for hematological surveillance of systemic indolent mastocytosis)	143

## Discussion

We describe a case of fatal hymenoptera venom anaphylaxis in a 41-year-old farmer suffering from systemic indolent mastocytosis, who had previously experienced non life-threatening anaphylactic episodes with hypotension and syncopes. By the time he was stung he had started but not completed a VIT. He had successfully carried out his first cycle of *Apis mellifera* VIT (8 sessions) and had started his first cycle of VIT (only 1 session) for *Polistes Dominulus*; despite the prompt use of an epinephrine injector, he had a serious anaphylactic reaction, followed by cardiovascular arrest and pulseless electrical activity (PEA) which necessitated long-lasting resuscitation attempts by the emergency medical team, who were on the scene within 20 min. Despite the re-establishment of cardiac contractility, he died in hospital 12 h after being stung. Death was due to post-anoxic brain damage, cardiovascular shock with terminal, multiorgan congestion, DIC and MOF, as confirmed during the autopsy. While the patient was undergoing inotropic and vasoactive amine infusion and ECMO support in the ICU, some blood samples were drawn from the distal extremity of the pulmonary catheter and stored in a laboratory because the patient was initially considered to be a potential organ donor (post-mortem retrieval of these biological samples revealed central blood serum tryptase levels of 8955 ng/mL); blood samples drawn 6 days after death, at autopsy, showed serum tryptase levels in central blood (4977 ng/mL) and peripheral blood (319 ng/mL) which were consistent with anaphylactic death and suggested that the farmer was a responder to VIT for *Apis Mellifera* VIT (IgE 0.44 kUI/L) but not for *Polistes Dominulus* (IgE 3.13 kUI/L) yet.

In association with the autopsy findings (which are typically non-specific in anaphylactic fatalities and can equally be caused by post-anoxic brain damage, cardiovascular shock, DIC and MOF) and circumstantial data, the laboratory results were crucial in understanding this case of fatal anaphylaxis, which is unusual in the following ways:

### The fact that central blood samples were drawn when the patient was still alive were available

Coroners and forensic pathologists are aware that, in cases of anaphylactic death, data on the serum tryptase levels of living patients are not normally demanded by clinicians during acute phases and so are not available for post-mortem comparison; during emergency situations, in cases of life-threatening anaphylaxis, the standard laboratory procedures used to detect and quantify tryptase and IgE take a relatively long time and are not routinely performed in acute pre-mortem phases because they are considered of no help in emergency clinical diagnosis and treatment [1, 2]. This is not in line, however, with the recommendations found in various guidelines such as those published by the Working Group of the Resuscitation Council UK 2008 regarding the surveillance of tryptase levels; these recommend drawing an initial blood sample as soon as possible after first aid or resuscitation has started, followed by serial samples (1–2 h and 24 h later) [27]. These recommendations were made because tryptase levels rise 15–30 min following the onset of anaphylaxis, peak within 2 h and fall back to the baseline within 24 h, and so their acquisition is a crucial step in the diagnosis of anaphylaxis in a living patient as well as in determining the correct therapy and follow-up; in any case, since tryptase levels may increase in the post-mortem phase for reasons unrelated to anaphylaxis (length of post-mortem interval, hemolysis/cytolysis, resuscitation procedures, acute cardiovascular diseases, bacterial/viral infections, sepsis, SIRS, ARDS, drug overdose, sudden infant death syndrome and mastocytosis), we consider that in cases of suspected anaphylactic death where there are most commonly no witnesses, the consultation of any pre-mortem serum tryptase levels could be pivotal [1, 2, 27–30].

### Evidence of relevant high pre-mortem (8955 ng/mL) and post-mortem (4977 ng/mL) central blood serum tryptase levels

The levels found in this case were unusually high and may have been caused by:

- the basic over-proliferation and dysfunction of mastocytes due to systemic indolent mastocytosis which causes an abnormal release of vasoactive mediators during an anaphylactic crisis.
- the fact that the patient was attached to an ECMO device which has pumps which can cause the mechanical damage of blood cell and lysis of basophils and eosinophils, which normally also store mature and enzymatically active tryptase.
- the presence of significant serum levels of IgE antibodies against *Polistes Dominulus* (IgE 3.13 kUI/L) able to interact, through antigen-IgE complexes, with a high and/or dysfunctional number of FcεgE receptors in mastocyte membranes [5, 9–11, 14–18, 20, 22–25, 31–34].

### Evidence of high post-mortem peripheral blood serum tryptase levels (319 ng/mL) in samples taken during autopsy 6 days after death

Such a high level in both central and peripheral blood resulted from the rise in mastocyte population that occurs in people with systemic indolent mastocytosis, whereby the degradation and clearance processes which are typically active during the post-mortem phase may have further increased the levels of this analyte. In a clinical setting, tryptase levels in living patients usually return to baseline within 24 h of the onset of an anaphylactic crisis, while the decline in post-mortem tryptase levels is slower and may persist for several days under the effects of post-mortem mastocyte lysis [1, 2, 28–30, 41].

### Evidence of high pre-mortem serum IgE against Polistes Dominulus

Unfortunately, at the moment the farmer was stung he had started but not completed venom immunotherapy treatment (VIT), having just finished the first cycle (8 sessions) of VIT treatment for *Apis Mellifera* venom but having only just started the first cycle (1 session) for *Polistes Dominulus* venom so that the serum IgE levels against this wasp were still high due to incomplete desensitization; this suggests, by exclusion, that the insect involved was most probably a wasp [41]. 

### The presence of systemic indolent mastocytosis and previous anaphylactic episodes indicate a high risk of serious anaphylaxis

As indicated in various publications the patient was at a very high risk of developing anaphylaxis because he was a male suffering from systemic indolent mastocytosis, with previous anaphylactic episodes involving hypotension and syncope, with baseline serum tryptase > 20  ng/mL and baseline serum total IgE compatible with immunological sensitization and an absence of urticaria pigmentosa [22, 35, 41].

### Drawing post-mortem peripheral venous blood samples using a sampling method described by garland et al

A number of authors including Garland et al. have pointed out that post-mortem serum tryptase levels are sample-site dependent and are higher in samples taken from heart blood; this is believed to be the result of tryptase spreading back from the heart and other mast cell-rich organs such as the liver as cell lysis takes place (both in the immediate post-mortem phase and during the ensuing hours) [27]. 

Therefore, the sample used for checking serum tryptase levels was drawn from the iliac vessels using an innovative procedure, which involved clamping the iliac vessels in order to prevent interference caused by analytes spreading in the back flow of blood from the central to the peripheral vessels. In short, blood samples for post-mortem analysis of serum tryptase levels should be taken as soon as possible, from clamped iliac and/or femoral veins and clinicians should be trained to draw, freeze and store blood during resuscitation procedures or immediately post mortem, in cases of suspected or diagnosed anaphylaxis, in order to obtain reliable immuno-allergological data before the onset of post-mortem cytolysis and other thanatological processes [1, 2, 27, 36–38, 40].

To summarize, this article focuses on the unusual features and medico-legal diagnostic procedures involved in the anaphylactic death of a male farmer with systemic indolent mastocytosis and sensitization to hymenoptera venom, who was stung by a hymenopteran, most probably a wasp. Rigorous inspection of the death scene, together with autopsy findings, immuno-allergological data and the victim’s medical history, all overseen by a multidisciplinary approach involving forensic pathologists, clinical pathology experts and immuno-allergologists, were crucial in determining not only whether the death was caused by hymenoptera venom but also to exclude medical liability in the way preventive VIT therapies and resuscitation procedures were performed.

Peripheral blood sampling from clamped iliac/femoral vessels, as soon as possible after death, should be a standard procedure during forensic investigations of anaphylactic deaths in order to avoid unreliable serum tryptase data resulting from:

- the effects of post-mortem cytolysis also involving mastocytes.
- thanatological and autolytic processes.
- the back flow of analyte (released from local pulmonary and heart-resident mastocyte populations) from central to peripheral blood.

Clinicians should also be trained to draw blood samples while resuscitation is ongoing in order to be able to make useful diagnostic comparisons of serum tryptase levels during the post-mortem phase of the investigation and can use the inguinal region [1, 2, 26, 27, 36–41].

In any case, the medico-legal post-mortem diagnosis of anaphylaxis, and not only in cases of patients suffering from mastocytosis, should not be made on the basis of tryptase alone but must be contextualized within clinical history, autopsy findings and ancillary tests, such as specific IgE [1, 2, 27, 36–38, 40, 41].
